# Recognizing Agricultural Headwaters as Critical Ecosystems

**DOI:** 10.1021/acs.est.3c10165

**Published:** 2024-03-04

**Authors:** Magdalena Bieroza, Lukas Hallberg, John Livsey, Laura-Ainhoa Prischl, Maarten Wynants

**Affiliations:** Department of Soil and Environment, Swedish University of Agricultural Sciences, Box 7014, 75007 Uppsala, Sweden

**Keywords:** Agricultural land use, stream networks, hydrology, stream chemistry, stream ecology

## Abstract

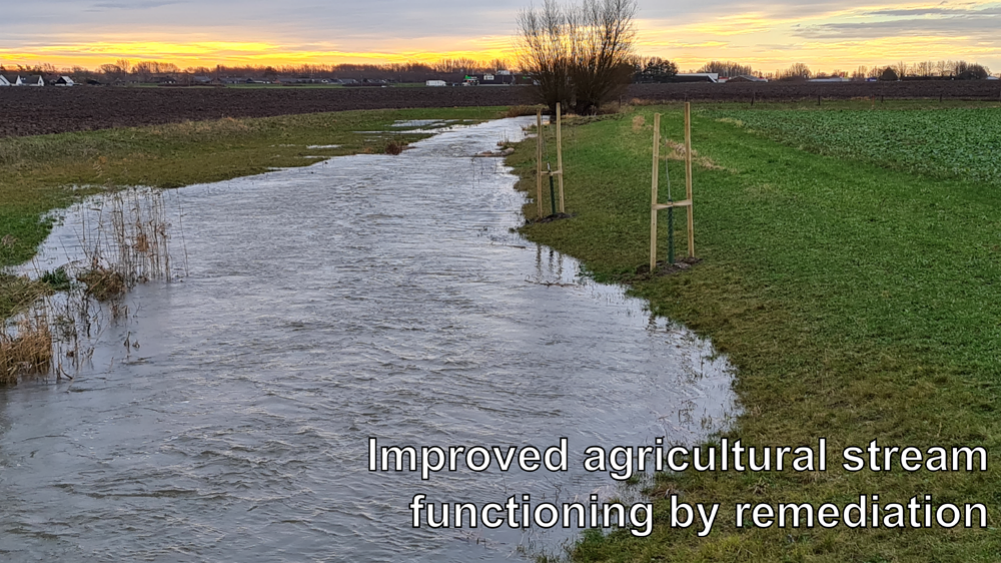

Agricultural headwaters are positioned at the interface between terrestrial and aquatic ecosystems and, therefore, at the margins of scientific disciplines. They are deemed devoid of biodiversity and too polluted by ecologists, overlooked by hydrologists, and are perceived as a nuisance by landowners and water authorities. While agricultural streams are widespread and represent a major habitat in terms of stream length, they remain understudied and thereby undervalued. Agricultural headwater streams are significantly modified and polluted but at the same time are the critical linkages among land, air, and water ecosystems. They exhibit the largest variation in streamflow, water quality, and greenhouse gas emission with cascading effects on the entire stream networks, yet they are underrepresented in monitoring, remediation, and restoration. Therefore, we call for more intense efforts to characterize and understand the inherent variability and sensitivity of these ecosystems to global change drivers through scientific and regulatory monitoring and to improve their ecosystem conditions and functions through purposeful and evidence-based remediation.

## Abstract 2

Agricultural headwaters are conduits of pollutants off the landscape, impeding improvements in water quality and ecology of downstream ecosystems, yet remain understudied.

## Introduction

**Figure 1:**
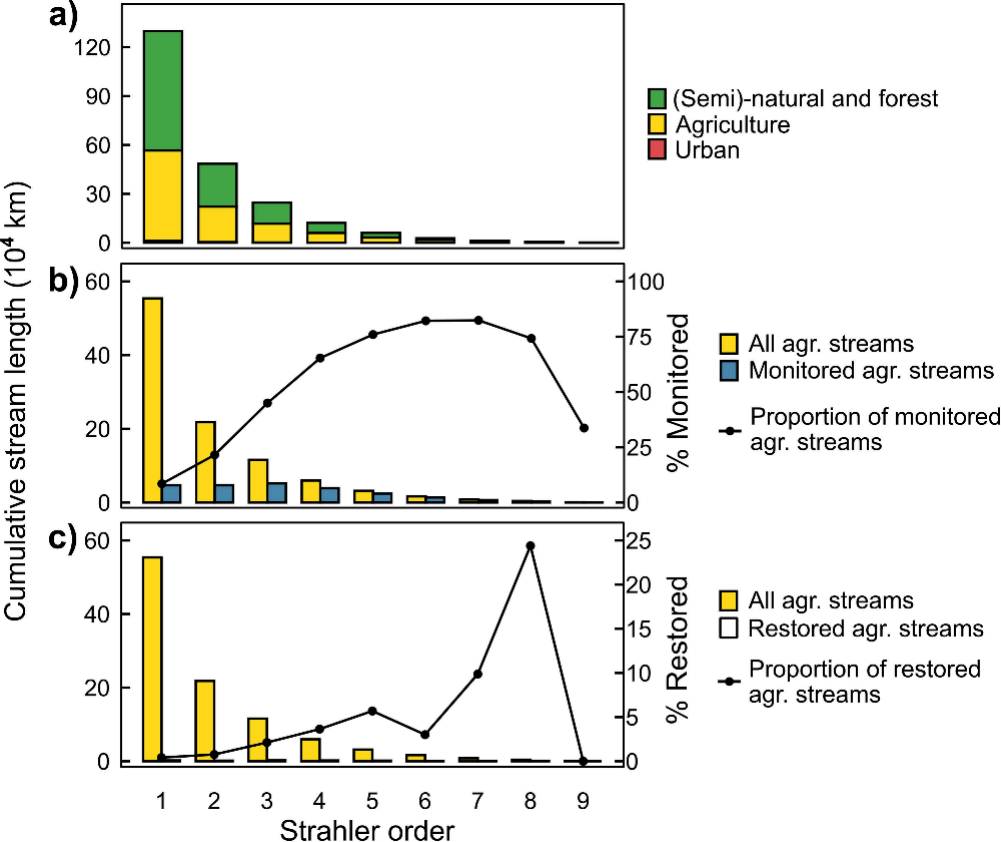
**a)** Cumulative length of European streams by the Strahler order, sorted by dominant catchment land use (the largest contribution of a given land use type): natural (forest and seminatural areas), agriculture, and urban.^2^ Comparison between the length of agricultural streams and **b)** monitored agricultural streams^3^ reported to the European Commission under the Water Framework Directive (WFD) and **c)** restored agricultural streams.^4^

Agricultural headwaters are considered 1st-2nd Strahler order streams draining agricultural landscapes that within the temperate climatic zone in North America and Europe correspond to around 50% of the stream length (Figure 1a). Agricultural headwaters comprise perennial and intermittent streams^1^ with a close coupling to the agricultural land they drain. Thus, unlike more natural streams, they are strongly influenced not only by hydrological, biogeochemical, and phenological cycles but also by the agronomic calendar. As the first link between terrestrial and aquatic environments, agricultural headwaters are subjected to diffuse pollution from agricultural soils that can deliver high loads of nutrients, sediments, pesticides, and other pollutants. To promote efficient drainage, agricultural headwaters are often subjected to significant geomorphological modifications such as straightening and channelization and periodical disruptive management practices such as dredging or vegetation removal. This not only alters their hydrological, biogeochemical, and ecological functions but also has cascading effects on all downstream ecosystems.

**Figure 2:**
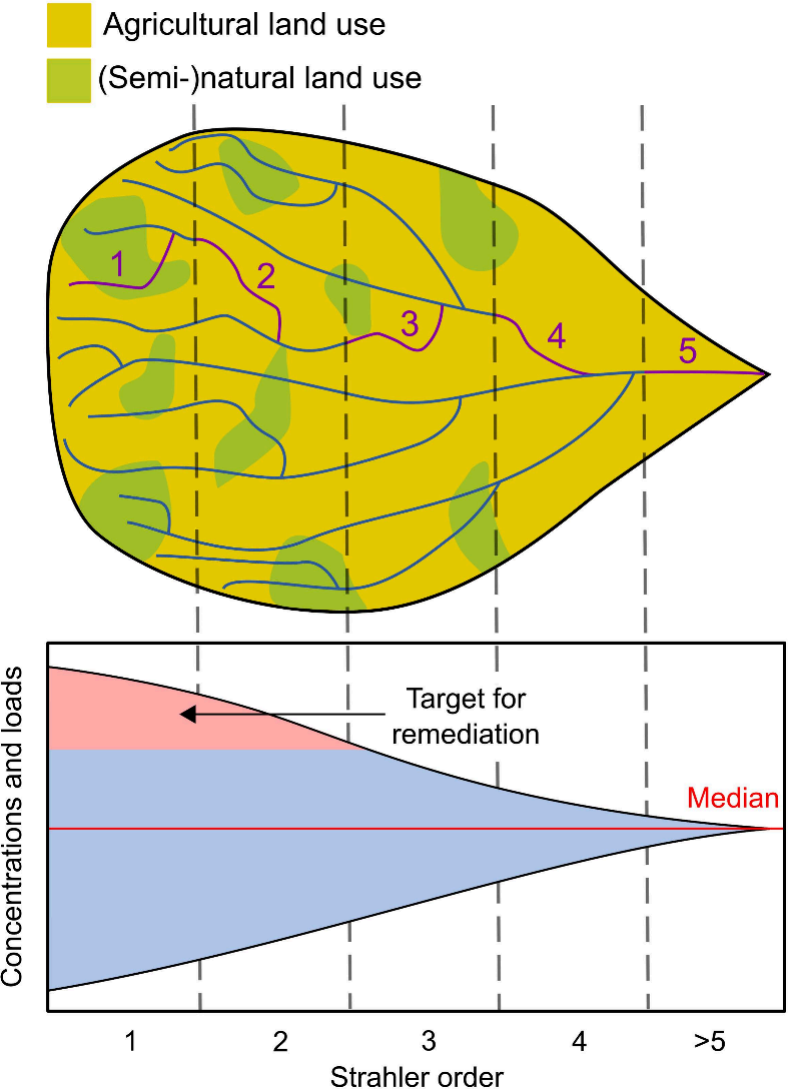
Variability in hydrological and biogeochemical functions is the highest in headwaters and is expressed in large variation in reported data on discharge, concentrations, and loads for solutes and particulates,^13,14^ diversity in concentration-discharge relationships,^15,16^ and greenhouse gas emissions.^17^ This variability results from large spatial and temporal heterogeneity in bedrock, soil texture, land use/land cover/land management, and stream corridor and channel properties. Since some of the highest pollutant concentrations, loads, and gas emissions are observed in agricultural headwaters, identifying these high extremes can help to target critical headwater agricultural catchments for prioritizing BMPs and stream remediation. This targeted remediation can help to improve not only the function of individual agricultural headwaters but also the function of entire downstream networks.

Despite this unique landscape position, their predominance,^5^ and important role in regulating water, elemental, and energy fluxes between terrestrial and downstream ecosystems, agricultural headwaters are understudied and undervalued as critical providers of ecosystem services. For example, agricultural headwaters are underrepresented in the European regulatory monitoring for chemical and ecological status^6^ (Figure 1b) and restoration and remediation efforts^7^ (Figure 1c). In the US, legal interpretation of what constitutes headwater streams under the Clean Water Act has restricted the extent of their restoration and management.^8^ Agricultural headwaters are also lacking regulatory protection within other international policies e.g., in China.^9^ As a result, most stream restoration interventions focus on treating downstream symptoms in larger rivers (Figure 1c). In headwater catchments, Best Management Practices (BMPs) and edge-of-field practices and structures such as buffer strips and wetlands are increasingly implemented to reduce primary pollution from agricultural land use. However, their observed impact on water quality and ecology in agricultural headwaters and downstream ecosystems is often unsatisfactory.^6,10^ These mixed results of land management interventions show the need for embedding the restoration of agricultural headwater streams into catchment remediation. While headwater stream restoration could reduce mobilization of secondary pollution accumulated in their corridors and improve their conditions and functions, it is rarely included in catchment management plans. Beside monitoring and restoration, scientific disciplines also tend to focus on larger water bodies, which has led to gaps in our understanding of the role of agricultural headwaters and their catchments in the transport and transformation of water, nutrient, and energy fluxes to downstream ecosystems. For example, aquatic ecology focuses on more pristine and larger water bodies, largely ignoring the ecological value and services that can be provided by agricultural headwaters.^11^ Likewise, hydrology and hydrochemistry often focus on large-scale land-water interactions, not capturing the heterogeneity of agricultural headwaters and their catchments.^12^ Overall, the lack of scientific focus together with monitoring gaps limit our understanding of underlying drivers of the large variability in hydrological and biogeochemical functions observed in agricultural headwaters (Figure 2), and this hinders identification of the best strategies to remediate and restore the function of agricultural headwaters.

Recognizing both the importance of agricultural headwaters and their overlooked position in scientific, monitoring, and restoration programs, we propose a holistic viewpoint for assessing their value by showcasing their key role in regulating water flows, water pollution, greenhouse gas (GHG) emissions, and biodiversity. We argue that in cascading river systems, agricultural headwaters and their catchments should not only be treated as the root cause of multiple problems (e.g., flooding, eutrophication, and habitat degradation) but also recognized as an essential cure when included in restoration and remediation efforts. Redefining agricultural headwaters could aid long-term and sustained environmental improvements as envisaged by the UN Sustainable Development Goals and regional water regulations (e.g., US Clean Water Act, EU Water Framework Directive, and European Green Deal).

## Agricultural Headwaters Regulate Flow Variability

Many of the challenges related to the hydrology of agricultural headwaters are shared with headwaters in general, but the significance of these factors is amplified within agricultural catchments. Headwaters make up the majority length of river networks (Figure 1a) and supply over half of the annual water volume entering higher order rivers.^8,18^ The hydrological signature of stream networks is shaped by headwater catchments that regulate storage and residence times of water.^8^ Due to their immediate connection to the contributing landscape, the hydrological response of agricultural headwaters can vary significantly within the same river network. Headwater streamflow variability is exacerbated in agricultural areas, leading to high flow amplitudes and intermittent or discontinuous flows.^19^ To enable crop production, hydrological processes in agricultural soils and headwaters were significantly modified. Installation of surface and tile drainage systems has increased the drainage rates of soils, while deepening and channelization of the stream network have promoted rapid downstream transport of water. Through this systematic increase in hydrological connectivity, agricultural headwaters and their catchments have lost most of their storage capacity to buffer water and nutrient fluxes from agricultural land. This has moved them toward more flashy hydrological regimes, with large variation in discharge on annual, seasonal, and storm event bases.^20^

Agricultural headwaters function as control points^21^ for downstream hydrological connectivity. This recognition is particularly important when considering the ongoing and future effects of climate change, which is projected to significantly alter precipitation distribution in time and space and increase the occurrence of extreme floods and drought.^22^ Moreover, seasonal redistribution of precipitation is predicted to lead to wetter winters in the temperate zone while simultaneously inducing more frequent plant water stress conditions during the growing season. This dual and opposing demand for irrigation during drought and drainage during flooding events poses a significant challenge to land and water management. Consequently, agricultural headwater catchments and streams will be at the frontline of climate change adaptation. Catchment water storage can be increased through mitigation measures, such as ponds, wetlands, or controlled drainage. In agricultural headwaters, there is a scope to adapt bed roughness through vegetation management, remeandering, or floodplain construction that can effectively regulate in-channel water velocity and residence times, dampen rainfall-runoff response,^23^ and provide additional ecological and water quality benefits.^24^

## Agricultural Headwaters Control Water Quality

The water quality signature of entire stream networks is generated in ubiquitous headwater catchments.^8,14^ At the same time, modifications to headwater geomorphology and diffuse pollution associated with agricultural land use are responsible for the widespread failures to reach improved chemical and ecological status in waterbodies.^25^ Thus, agricultural headwaters and their catchments are ecosystem control points^21^ of stream networks, contributing significant loads of nutrients, suspended sediments, and other pollutants (e.g., pesticides, pharmaceuticals, microplastics) derived from agricultural activities.^26^ Despite common water quality pressures and similar land use trajectories within temperate areas,^10^ agricultural headwaters vary significantly in terms of water quality reflecting large spatial and temporal heterogeneity in the land-water interactions and land management.^12,14^ This high hydrochemical variability is expressed for example in diverse concentration-discharge relationships observed for nutrients, carbon, and sediments in agricultural headwaters, varying from chemodynamic to chemostatic in contrast to high order streams with predominantly chemostatic slopes.^15,16^ This variability results from variation in the way agricultural catchments are managed and how they modulate and transport solutes and sediments. The common driver is the long-term accumulation of legacy nutrients, in agricultural soils, saturated and unsaturated zones, and within bed sediments of headwater streams,^10^ but agricultural catchments and streams (riparian and hyporheic zones) can have varying pollution buffering capacity.^27^ The continuous release of legacy nutrients into aquatic environments, together with higher occurrence of extreme hydrological events, can control water quality in the long term and override positive effects of BMPs and catchment remediation.^28−30^ Thus, agricultural headwaters capturing secondary and legacy pollution are one of the key points of intervention to focus remediation measures such as constructed/reconnected floodplains and remeandering. Reported solute and sediment retention rates during low-to-medium magnitude flow conditions^24,31,32^ in remediated agricultural headwaters are within similar order of magnitude compared to values reported for the edge-of-field buffer strips and wetlands.^33^ Thus, remediation of agricultural headwaters not only can improve their function but also have cascading impacts on water quality and ecology of downstream ecosystems.^6,10,24,31^ However, restoration of agricultural headwaters is underrepresented in management compared to catchment remediation (e.g., edge-of-field wetlands) and restoration of larger rivers (Figure 1c). This together with knowledge gaps related to the functioning of agricultural headwater catchments and streams has led to poor and slow water quality improvements and growing skepticism among stakeholders implementing BMPs and catchment remediation measures.

## Agricultural Headwaters Are Hot Spots for Gas Emissions

Inland watercourses are increasingly recognized as important contributors to the global GHG budget and consequent global radiative forcing, contributing to 5% carbon dioxide (CO_2_), 4% of nitrous oxide (N_2_O), and 9% of methane (CH_4_) global anthropogenic emissions.^17,34^ Streams are consistently supersaturated with GHG, and the combined CO_2_ equivalent of these emissions may even offset the global terrestrial carbon (C) sink.^35^ Thus, agricultural headwaters are hot spots of GHG emissions that disproportionately influence global fluvial emissions. Their high hydrological connectivity not only promotes instream GHG production by supplying nutrients, labile carbon, and sediments but also mediates transfer of terrestrially produced GHG from agricultural soils.^36,37^ However, large-scale GHG inventories often underrepresent agricultural headwaters spatially by focusing on capturing variability across diverse ecosystems and temporally by measuring predominantly during baseflow conditions.^36^

Headwaters are critical for global C cycling and thereby CO_2_ emissions, accounting for 36% of all CO_2_ emitted from running waters.^17^ These emissions stem from direct instream mineralization of organic C and indirect terrestrially produced CO_2_, with the inputs of organic and inorganic C being the highest in headwaters. Hydrological connectivity in headwaters enhances indirect CO_2_ emissions,^38^ which are particularly high from artificially drained agricultural headwater catchments with highly productive soils.^37,39^ Stream N_2_O emissions are tightly linked to agricultural production, promoted by microbial denitrification and nitrification under elevated nitrate concentrations.^35^ Nitrogen fertilization of agricultural crops explains 45% of N_2_O emissions from global watercourses.^34^ As with CO_2_, a considerable fraction of N_2_O emissions from agricultural headwaters also originates from indirect sources and subsurface pathways that can dominate total stream emissions.^40^ Although CH_4_ production represents a negligible fraction of total C fluxes from streams, CH_4_ emissions from watercourses can be substantial, amounting to half of the combined emissions from wetlands and lakes.^41^ In an agricultural context, there is a relative scarcity in CH_4_ studies compared to other GHG and thus greater uncertainty surrounding the magnitude and controls of CH_4_ emissions. In addition, estimates of CH_4_ emissions rely heavily on diffusive measurements, largely overlooking the contribution of CH_4_ from ebullition, which can be substantial during episodic events. Deposition of fine sediments has consistently been reported as a key driver of CH_4_ production^42^ suggesting that low-gradient and fluvially unstable agricultural headwaters prone to erosion can support methanogenesis by providing organic matter-rich material and anoxic conditions. From a management perspective, the challenge of mitigating indirect GHG emissions has to be addressed with broader approaches, that integrate traditional stream mitigation measures (e.g., buffer zones, floodplains, and channel impoundments) with in-field measures that also target the landscape source and delivery of GHG.^43^

## Agricultural Headwaters Shape Ecosystem Structure and Function

As ecological habitats, agricultural headwaters are home to a specialized subset of fauna and flora adapted to the seasonally changing flow and nutrient conditions.^44^ Agricultural headwaters and their riparian zones can function as corridors within agricultural landscapes. However, human alterations to agricultural headwaters and their catchments through fluxes of nutrients and sediments and the physical alteration of stream channels and their riparian zones have negative effects on community composition and ecosystem function.^45^ For example, agricultural land use can increase stream ecosystem productivity^46^ due to removal of riparian shading, shifting energy sources toward autochthonously derived carbon.^47^ To improve our understanding of underlying consumer dynamics, there is a need to further link metabolic regimes to food web ecology for predicting food web structure from stream energetics.^48^ Differences in community composition and functioning between agriculturally impacted and natural streams cannot solely be explained by anthropogenic activities but are also influenced by differences in underlying topography and soil texture^49^ in their catchments. The distinctive geomorphology within agricultural catchments is often not accounted for in ecological and chemical assessments, leading to an arbitrary comparison of agricultural headwaters to seminatural reference streams.^50^ Given the inherent landscape differences between agricultural and natural headwaters and the pervasive impact of nutrient legacies, we therefore argue that there is a need to develop specific reference thresholds for evaluating agricultural streams.^7^ Instead of changing the assessment criteria, agricultural headwaters are often excluded from basin-scale action plans altogether.^7^ From a management perspective, agricultural headwaters are often in private land ownership and vital for the agricultural services they provide, e.g., soil drainage, to enable crop production. By ignoring this multifunctionality of agricultural headwaters, we are setting up restoration and remediation activities for failure and potentially increasing the divide between nature conservation and landowners.^51^

## Recognizing the Role and Importance of Agricultural Headwaters

Agricultural headwaters are everywhere but at the same time much overlooked, despite their important role in regulating hydrological, chemical, and ecological functions and quality of downstream ecosystems. They are typically transformed into passive pipes transporting rapidly agricultural pollutant loads, but with improved management, they could become stream ecosystems that actively regulate water, matter, and energy fluxes.^6,11,16,52^ As agricultural headwater catchments and streams are currently lacking buffering capacity to regulate accelerated water and biogeochemical fluxes, they are extremely sensitive to global change impacts.^53^ Global change is going to exacerbate existing challenges in agricultural headwaters. Many agricultural headwaters can seasonally dry out, shifting their regimes from perennial to intermittent conditions^1^ with major consequences for their biogeochemical and ecological functions.^28,54^ Higher frequencies of extreme hydrologic events are forecast to increase fluxes of nutrients and sediments^29^ and GHGs.^37^ Therefore, a paradigm shift is needed beyond the current view of agricultural headwaters as mere conduits for excess water and pollutants. Instead, we should recognize them as critical ecosystems and interfaces between terrestrial and aquatic environments and intensify the efforts to study, monitor, and restore them.

Agricultural headwaters should not be treated as outliers but rather as an equal part of a wide spectrum of aquatic ecosystems. We urge the scientific community to describe their inherent hydrological, geomorphological, biogeochemical, and ecological variability and policy makers to incorporate this variability into existing evaluation and classification frameworks. New measurement and valorization techniques are needed that can be applied to both agricultural and natural headwaters. For example, existing approaches to describe and quantify ecological status are aimed at gravel-bed streams, and there is a lack of equivalent approaches for agricultural headwaters with fine bed sediments.^55^ In the same manner, measurements of nutrient uptake velocities rely on nutrient additions to increase concentrations above background level,^46^ which is extremely difficult and costly to achieve in agricultural headwaters. Novel interdisciplinary measurement approaches could build on cutting edge technologies that are increasingly available, such as in situ sensors and environmental DNA, that can be deployed in different types of aquatic systems.^56^ Strategically distributed networks of such sensors can help to characterize the large spatial and temporal variability in the hydrological, biogeochemical, and ecological functions of agricultural headwaters, improve process understanding of differences in how headwater agricultural catchments accumulate and release solutes and pollutants, and identify stream networks’ control points for targeting monitoring, management, and remediation. A fusion of experimental and modeling approaches would be needed to establish an optimal and cost-effective number of monitoring points in agricultural headwaters to capture variability in water quality both for scientific and regulatory purposes, e.g., to supplement existing monitoring networks. Finally, agricultural headwaters and their catchments should become an integral part of highly instrumented experimental catchment networks for monitoring long-term ecosystem change, such as Long-term Ecological Research (LTER), the National Science Foundation’s National Ecological Observatory Network (NEON), and Critical Zone Observatories: Research and Application (OZCAR), Terrestrial Environmental Observatories (TERENO), and Swedish Infrastructure for Ecosystem Science (SITES), as they are currently severely underrepresented. UK Demonstration Test Catchments (DTC)^57^ and Irish Agricultural Catchment Programme (ACP)^30^ are great examples of long-term monitoring in agricultural headwater catchments that characterize agricultural impacts and facilitate knowledge exchange with local stakeholders.

Improved understanding of function variability in agricultural headwaters is critical not only to establish underlying mechanisms and improve regulatory monitoring but also to identify cost-effective ways to restore and remediate agricultural headwaters and their catchments so both headwaters and downstream ecosystems function better. From a management perspective, the challenge of mitigating pollution in agricultural headwaters must be addressed with broader approaches that integrate traditional farm- and field-based BMPs, e.g., optimized fertilization and cover crops, edge-of-field practices, and structures with restoration and remediation of streams through remeandering, widening, or floodplain reconnection or reconstruction. Remediation of agricultural headwater streams is the missing link between catchment remediation and larger river restoration. It offers great potential for synergies between different ecosystem functions, such as flood/drought, nutrient and biodiversity regulation, and better overall cost-effectiveness and potential to achieve several policy goals simultaneously^6,53^ e.g., climate adaptation and improvements in water quality and biodiversity. However, when evaluating success of restoration and remediation of agricultural headwaters, consideration should be given to their specific environmental and legacy constraints,^58^ and therefore, realistic goals and success measures should be set. We also urge scientists and stakeholders to communicate and consider differences in effectiveness between catchment vs stream remediation measures. As in-field and edge-of-field measures target mostly primary pollution sources, their apparent effectiveness is higher compared to in-stream remediation targeting not only primary but also legacy and secondary sources.^28^ As improvements in stream ecosystem function are slow and unsatisfactory, we need to combine catchment and stream remediation^6,10^ and intensify studies on how to target and design measures for best cost-effectiveness and understand why the same measure can have a different impact in different catchments and streams. Here, further progress can be achieved by combining high-spatial and high-frequency measurements and experimental data with stream and catchment models.^59^ Given the diversity of agricultural headwater catchments, there is a need for bottom-up and local community-led approaches for management, restoration, and remediation that can stimulate knowledge exchange between scientists and stakeholders. To this end, the authors of this paper have been supporting with monitoring and feedback the catchment and stream remediation project driven by a farming association in Tullstorpsån and Ståstorpsån,^60^ which is an excellent example of how such initiatives should be planned and executed. This knowledge exchange is particularly needed to anchor restoration and remediation efforts with scientific evidence of their planned and observed effects and secure support and engagement from local farming communities.

## Implications

Scientists, authorities, and stakeholders have the power to transform agricultural headwaters from passive pipes to active stream ecosystems, realizing their full hydrological, biogeochemical, and ecological functions. This can be achieved through intensified and joint efforts to study, monitor, and remediate agricultural headwater catchments and streams, so that their important agronomic and drainage services finally reconcile with their ecosystem function. Improving this impaired function is critical, as agricultural headwaters are at the root of most stream networks and underpin freshwater quality and biodiversity. Therefore, further scientific and monitoring efforts are needed to better understand the complex links between land management and catchment function, controlling the large variability in water quality, gas emissions, and biodiversity in agricultural headwaters. This improved knowledge would provide much needed guidance for stream restoration, which, nowadays, is often based on stakeholder preferences and available funding rather than scientific evidence. As agricultural headwater catchments support livelihoods of farming communities, there is a need for continuous knowledge exchange and dialogue between stakeholders and scientists, which could, for example, be achieved through citizen science projects supporting regulatory and operational monitoring. As global change exacerbates negative impacts on terrestrial and aquatic ecosystems in agricultural headwater catchments, this recognition and redefining of agricultural headwaters as critical ecosystems is both timely and imperative.
